# Molecular predictors of locoregional and distant metastases in oropharyngeal squamous cell carcinoma

**DOI:** 10.1186/1916-0216-42-53

**Published:** 2013-10-16

**Authors:** Brittany R Barber, Vincent L Biron, Alexander C Klimowicz, Lakshmi Puttagunta, David WJ Côté, Hadi Seikaly

**Affiliations:** 1 Division of Otolaryngology-Head and Neck Surgery, University of Alberta, 8440-112 Street NW, Edmonton T6G 2B7, Canada; 2 Department of Pathology, University of Alberta, 8440-112 Street NW, Edmonton T6G 2B7, Canada; 3 Department of Oncology, University of Calgary, 1331-29 Street, Calgary, Alberta T2N 4N2, Canada; 4 Functional Tissue Imaging Unit, Translational Research Laboratory, Tom Baker Cancer Center, 1331-29 Street, Calgary, Alberta T2N 4N2, Canada; 5 Section of Otolaryngology-Head & Neck Surgery, Department of Surgery and Oncology, University of Calgary, 1331-29 Street, Calgary, Alberta T2N 4N2, Canada

**Keywords:** Biomarker, Ki67, p16, p53, EGFR, Bcl-xL, Oropharyngeal, Cancer, Surgery, Radiation

## Abstract

**Background:**

The incidence of oropharyngeal squamous cell carcinoma (OPSCC) is increasing due to fundamental changes in oncogenesis related to effects of the human papilomavirus (HPV). Virally-mediated tumours behave and respond to treatment differently than their classic, carcinogenically-mediated counterparts despite similar stage and grade of disease. This difference in behaviour has lead to investigation of etiologies of OPSCC at the molecular level.

Molecular biomarkers offer potential insight into the behaviour of OPSCC. Identifying a subset of patients that are more likely to have recurrence and distant metastasis is valuable for prognostication and treatment planning. There is limited information regarding the profiles of these biomarkers in locoregional and distant metastases in OPSCC.

**Objective:**

This study was designed to identify biomarker profiles predictive of locoregional and distant metastases and recurrence in OPSCC.

**Methods:**

Cross-sectional study of a prospectively-collected oropharyngeal tumour database was undertaken. All patients with OPSCC presenting to the University of Alberta Hospital from 2002*-*2009 were included in the study. Data collection from the Alberta Cancer Registry, including demographics, nodal status, distant metastases, treatment, recurrence, and survival, was undertaken. Tissue micro-arrays (TMAs) were constructed for each tumour specimen using triplicate cores (0.6mm) of formalin-fixed, paraffin-embedded (FFPE) pre-treatment tumour tissue. TMAs were processed using immunohistochemistry for p16, EGFR, Ki67, p53, and Bcl-XL. Positivity for each biomarker was determined using quantified AQUAnalysis ® scores on histoplots. Multivariate statistics were utilized to assess the relationship between each biomarker and locoregional and distant metastases, as well as recurrence-free survival (RFS).

**Results:**

High expression of p16 (p=0.000) and Bcl-XL (p=0.039) independently demonstrated a significant association with nodal disease at presentation. Kaplan-Meier analysis demonstrated improved RFS in patients with high p16 and decreased RFS in patients with high p53 expression. Cox regression analysis supported p16 as an independent prognosticator for improved RFS. p53 demonstrated an association with recurrence, but when compared to p16 status, nodal status, and staging, was not an independent predictor of recurrence.

**Conclusions:**

Biomarker profiling using p16, Bcl-xL, and p53 may be useful in prognostication and treatment planning in patients with OPSCC.

## Background

Head and neck squamous cell carcinoma (HNSCC) is the sixth most common malignancy worldwide, with oropharyngeal squamous cell carcinoma (OPSCC) accounting for approximately half of these cancers. The major etiological factors associated with OPSCC are alcohol consumption, tobacco use, and human papillomavirus (HPV) infection. Carcinogenic and virally-mediated events induce numerous molecular and genetic alterations, causing abnormal molecular processes that eventually manifest as malignancies. The present treatment protocols for OPSCC are based on the anatomic extent of the disease (TNM staging) and do not take into account the ongoing biologic and molecular processes. Treatment protocols involving surgery, radiation, and/or chemotherapy are aggressive and may lead to significant toxicity, especially when used as combined modalities.

Molecular biomarkers are known to be useful in predicting survival outcomes for OPSCC patients. Oncogenic HPV infection is associated with upregulation of p16, a protein now widely utilized as a surrogate marker of HPV-positive disease [1,2]. Many authors have shown that HPV-positive oropharyngeal cancers are associated with significantly improved survival. Other molecular biomarkers including epidermal growth factor receptor (EGFR), B-cell lymphoma extra large (Bcl-xL), p53, and Ki67, have been shown to have prognostic significance^i^[3]. High EGFR expression has been correlated with poorer survival, while low levels of the anti-apoptotic protein, Bcl-xL, have been shown to be associated with improved response to radiation [4]. A recent study has suggested that elevated Ki67 levels may be associated with improved response to radiotherapy [5]. Despite the available data, there has been limited investigation regarding the role of different biomarker profiles in locoregional and distant metastasis of OPSCC. Identifying a subset of patients that are more likely to have recurrence and distant metastases is valuable when counselling patients about treatment options and prognosis, and making treatment decisions.

The purpose of this study was to identify specific biomarker profiles predictive of nodal disease at presentation, the development of distant metastases, and potential for recurrence in patients with OPSCC.

## Methods

Approval for the study was obtained from the University of Alberta Health Research Ethics Board (Pro00016426).

### Study population

The study population was comprised of 237 histologically-confirmed OPSCC patients diagnosed between 2002–2009 at the University of Alberta, Edmonton, Canada. All patients were treated with primary surgery, and further adjuvant treatment was determined by the final pathologic results. Demographic and clinicopathologic patient characteristics were collected from the Alberta Cancer Registry (ACR).

### Tissue microarray (TMA) construction

Tissue microarrays (TMAs) were constructed from pre-treatment tumour tissue. Patients with small pre-treatment biopsy specimens that would require sacrifice of the entire block were not included (accounting for approximately 20% of patients who were treated with non-surgical protocols). Formalin-fixed, paraffin-embedded (FFPE) tumour samples were retrieved for TMA construction. Hematoxylin and eosin-stained (H&E) slides were obtained from surgical specimens and reviewed by a Head and Neck pathologist to confirm the diagnosis of squamous cell carcinoma (SCC). Each TMA was assembled from duplicate or triplicate 0.6 mm cores randomly sampled from the tumour-containing area of each FFPE block. Five samples of normal tonsil tissue from patients with no history of HPV infection or OPSCC were included in the TMAs as reference samples to ascertain normal biomarker expression levels.

### Quantitative fluorescent immunochemistry

Each TMA was deparaffinized in xylene, rinsed in ethanol, and rehydrated. Heat-induced epitope retrieval for Ki67 was achieved by heating slides to 121 degrees Celcius in a citrate-based Target Retrieval Solution (pH 6.0) (Dako) for 6 minutes in a decloaking chamber. For Bcl-XL, the slides were bathed in an EDTA-containing Target Retrieval Solution (pH 9.0) (Dako) for 3 minutes, and each slide was stained for Bcl-XL using rabbit monoclonal anti-Bcl-XL (1:500 dilution, Cell Signalling). Rabbit Envision+ was used with tyramide-Cy3 (Perkin-Elmer) for Ki67, or tyramide-Cy5 (Perkin-Elmer) for Bcl-XL, to determine the expression of each biomarker in the TMA cores. A similar isolation technique was used for EGFR in accordance with previous reporting [6]. TMA slides were stained for rabbit monoclonal anti-EGFR antibody (1:1000 dilution, Epitomics) for 60 minutes. Rabbit Envision+ kit (DAKO) was used in conjunction with Tyramide-Cy5 (Perkin-Elmer) to visualize EGFR expression. p53 was isolated as previously published using mouse monoclonal anti-p53 (DAKO) [7]. p16 was isolated in a similar manner as has been previously reported [8]. p16 was also isolated using a commercially-available p16 antibody (Clone Jc8; Novus Biologicals, Littleton, CO). The intensity of p16 staining was scored using a 4-point (0–3) semi-quantitative scale (0=negative; 1=weak, <5%; 2=focally strong, 5-80%; 3= diffusely strong, >80%). Tissue from HPV-infected cervical intraepithelial neoplasia was used as the p16-positive control. The highest score amongst the 3 cores was used for analysis, and the tumour was graded as p16-negative (score 0–2) or p16-positive (score 3). Guinea-pig anti-pancytokeratin (PCK) antibody and an Alexa488 conjugated anti-guinea pig secondary antibody were used to identify the entire epithelial tumour compartment. Slides were mounted using Prolong Gold Anti-fade with DAPI (Invitrogen).

Each TMA was scanned by HistoRx™ PM-2000 and analyzed using AQUAnalysis® software (version 2.2.1.7). The PCK-positive region was denoted as the tumour compartment, the DAPI-positive region was denoted as the tumour nuclear compartment, and the Ki67 compartment area was defined as the Ki67 positive area inside the tumour nuclear compartment. Areas with less than 20% tumour tissue were determined to be insufficient for analysis and were excluded from the analysis (approximately 20% of non-surgical specimens). The AQUAnalysis® software compared each tumour core to normal oropharyngeal squamous tissue to establish a baseline expression value. AQUA scores representing the exposure time-adjusted pixel intensity density of biomarker proteins within the tumour compartment of each core were calculated and a quantified value of the expression of each biomarker was obtained for further analysis.

### Statistical analysis

All statistical analyses were performed using SPSS (Version 21.0). The expression value for each biomarker was qualified as positive based on current literature standards or, when there were no standards, values above the median for the cohort were qualified as positive. For p16, cores that expressed greater than 80% positivity on the histoplot generated by the AQUAnalysis® software were considered positive, as is consistent with current literature standards [9]. For EGFR, values that were more than one standard deviation below the mean were considered low. For p53, Bcl-XL, and Ki67, values that were above the median value calculated for the entire cohort of 226 specimens were deemed positive. A Chi-squared analysis was used to evaluate the association between the positivity of each biomarker and nodal disease at presentation, as well as the development of distant metastatic disease. Chi-squared analysis was also utilized to determine associations and clinical co-existence of biomarker expression. Kaplan-Meier analysis was used to assess 5-year recurrence-free survival (RFS) for each biomarker profile, and a Cox regression analysis was utilized to evaluate the prognostic value of each biomarker compared to the other biomarkers, and to known prognosticators of recurrence (stage, nodal status).

## Results

### Cohort characteristics

226 specimens were available for analysis and had adequate tumour tissue for generation of Histoplot analysis with AQUAnalysis® software. The average age of the patients was 58.1 years. The study population with adequate tumor specimens consisted of 177 males and 49 females. Nodal disease at presentation existed in 84% of the study population. 88.5% of patients had advanced-stage disease (Stage 3 or 4). All patients were treated curatively with multiple modalities including primary surgical or radiation treatment regimens. Median follow-up time was approximately 2.88 years. The recurrence-free survival (RFS) for the study population was 74.8%.

### Biomarker expression

**Figure 1:**
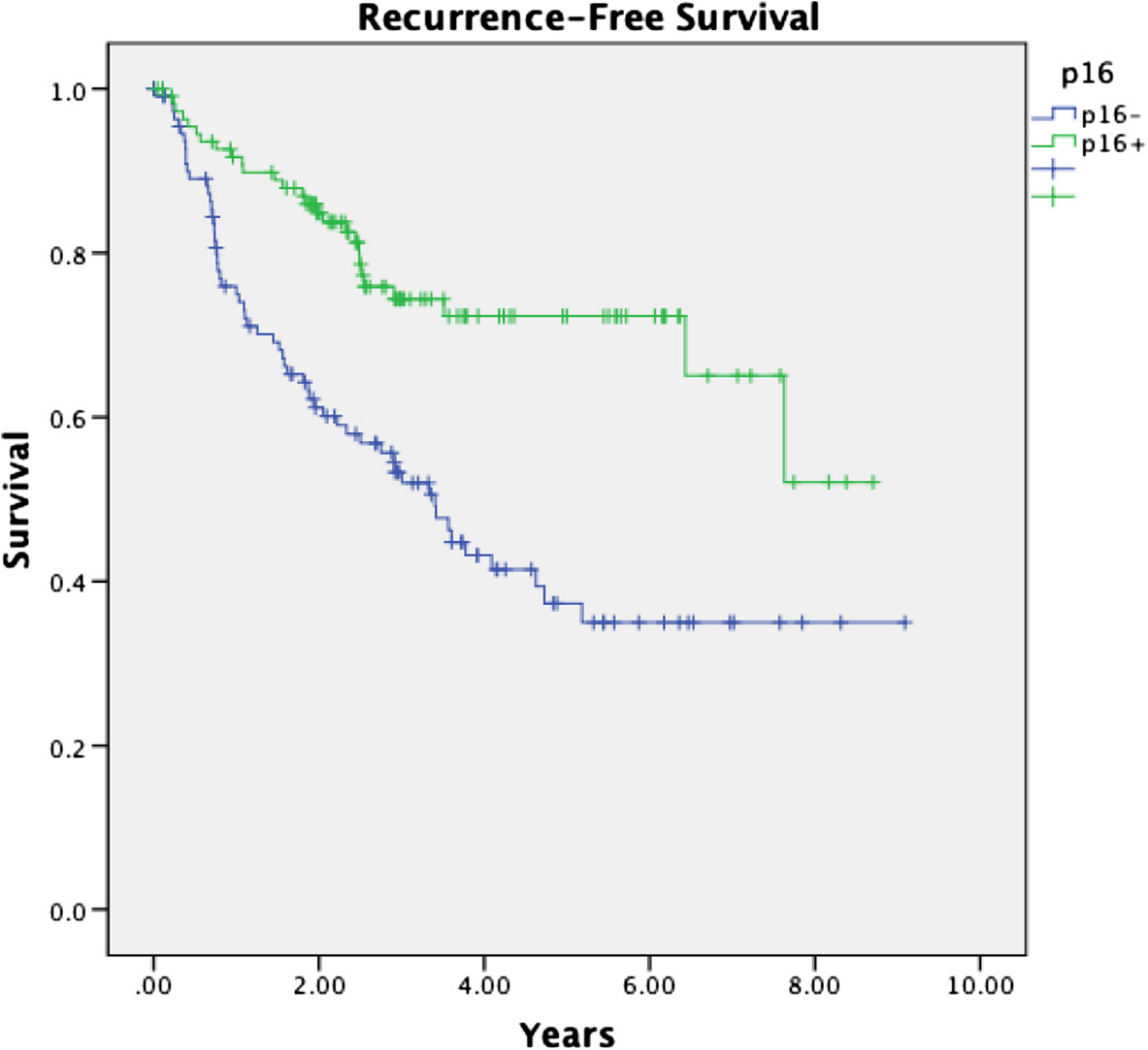
Kaplan-Meier analysis of p16 expression and RFS in OPSCC from 2002–2009 for all treatment modalities.

**Figure 2:**
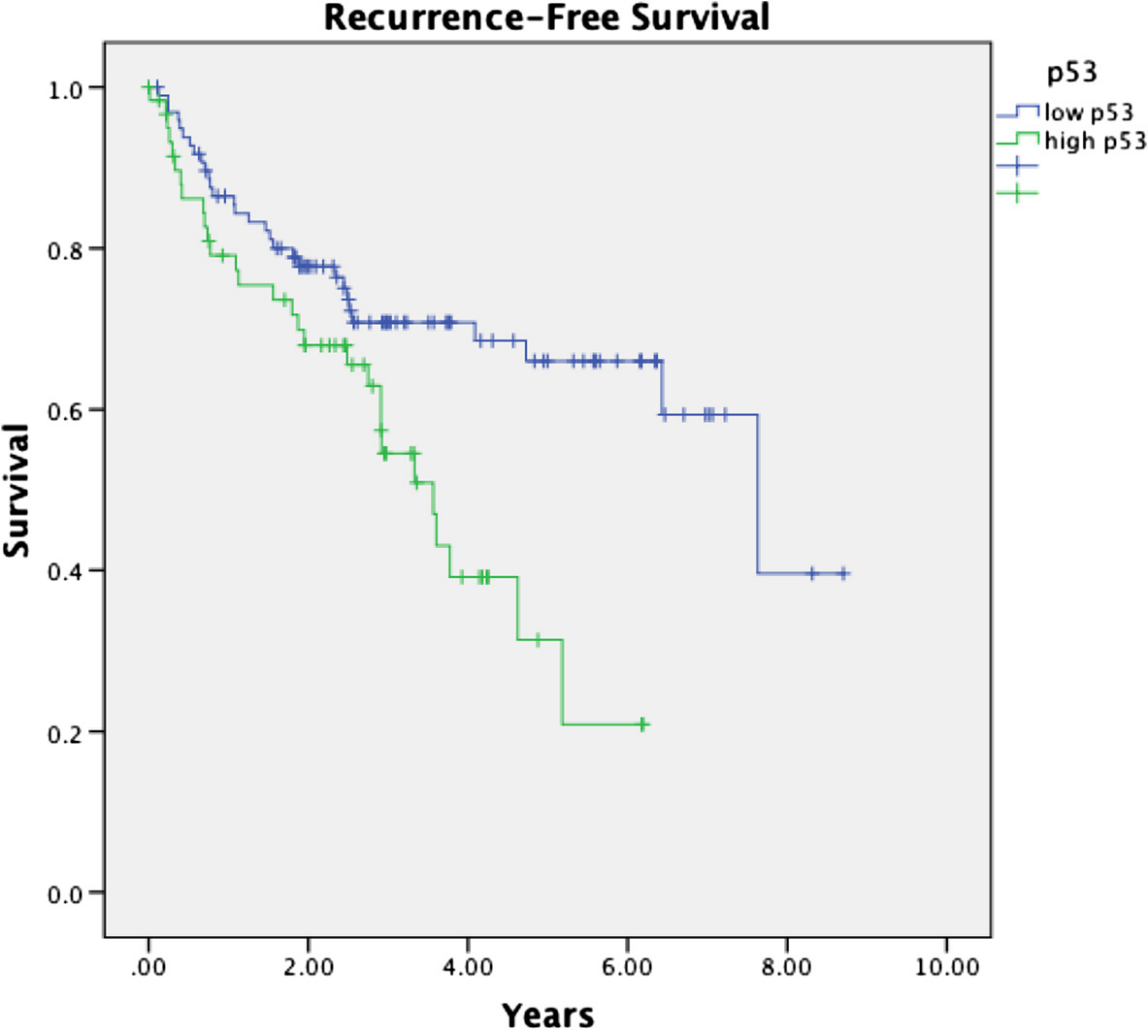
Kaplan-Meier analysis of p53 expression and RFS in OPSCC from 2002–2009 for all treatment modalities.

Chi-squared analysis demonstrating the relationship between biomarker expression and nodal disease at presentation as well as the development of distant metastases is demonstrated in Table 1. Kaplan-Meier analysis and relevant survival curves are demonstrated in Table 2 and Figures 1 and 2. Cox regression analysis comparing the effect of each biomarker on recurrence-free survival (RFS) both with (Table 3) and without known prognosticators of recurrence (Table 4) is also demonstrated.

**Table 1:** Chi-squared analysis of p16, Ki67, EGFR, p53, and Bcl-xL with nodal disease at presentation and development of distant metastases in OPSCC patients treated with all modalities from 2002–2009

***Biomarker***	***Nodal disease at presentation***		***Development of distant metastases***	
	***x***^***2***^	**p-value**	**x2**	**p-value**
**p16**	12.480	0.000**	0.225	0.748
**Ki67**	0.410	0.737	1.830	0.189
**EGFR**	0.870	0.427	0.793	0.508
**p53**	3.407	0.092	0.548	0.561
**Bcl-xL**	4.915	0.039**	0.113	0.852

**Denotes statistical significance.

**Table 2:** Kaplan-Meier analysis of biomarker expression and RFS in OPSCC patients from 2002–2009 for all treatment modalities

***Biomarker***	***Kaplan-meier analysis RFS*** **P-value**
**p16**	0.000**
**Ki67**	0.319
**EGFR**	0.097
**p53**	0.005**
**Bcl-xL**	0.147

**Denotes statistical significance.

**Table 3:** Cox regression analysis of biomarker expression with RFS for all treatment modalities in OPSCC patients from 2002–2009

***Biomarker***	***B***	***CI***	***P-value***
**p16**	-0.891	0.215-0.782	0.029**
**Ki67**	0.178	0.569-2.514	0.108
**Bcl-xL**	-0.312	0.392-1.369	0.231
**EGFR**	0.524	0.783-3.643	0.367
**p53**	0.330	1.033-3.765	0.021**

**Denotes statistical significance.

**Table 4:** Cox regression analysis of biomarker levels with RFS when compared to known prognosticators for recurrence (stage, nodal status)

***Biomarker***	***B***	***CI***	***P-value***
**p16**	-0.890	0.226-0.745	0.003**
**p53**	0.491	0.867-3.083	0.129
**Stage**	0.550	0.335-8.967	0.512
**Nodal status**	0.467	0.487-5.227	0.441

**Denotes statistical significance.

### p16 expression

p16 positivity was confirmed in 112 patients (48.7%). High p16 expression was correlated with nodal disease at presentation (p=0.000, Table 1), and this was substantiated by further univariate and multivariate analyses (p=0.001, p=0.049). Combined biomarker analysis revealed a significant inverse association between expression of p16 and p53 (p=0.003). Kaplan-Meier analysis revealed a significant recurrence-free survival (RFS) advantage with p16 positivity across all treatment modalities (p=0.000) (Figure 1). Further analysis demonstrated a significant increase in RFS with p16-positivity when compared to other biomarkers (p=0.029, Table 3) and when compared to known prognosticators for recurrence (p=0.001, Table 4).

### Ki67 expression

The typical staining pattern for Ki67 was predominantly nuclear as expected. 56.5% of the tumour specimens were found to be greater than the median calculated for the cohort (975.67), and therefore, Ki67-positive,. Chi-squared analysis revealed no significant association between Ki67 expression and nodal disease at presentation (p=0.737) or distant metastasis (p=0.189). Kaplan-Meier analysis demonstrated no difference in RFS with elevated Ki67 staining (p=0.319). No significant difference in RFS was noted when compared to other biomarkers (p=0.108) or when compared to known prognosticators for recurrence (p=0.165).

### EFGR expression

High expression of EGFR was confirmed in 84.1% of patients. There was no association between high EGFR expression and nodal disease at presentation (p=0.427) or development of distant metastatic disease (p=0.508). Kaplan Meier analysis demonstrated no effect on RFS. Cox regression analysis revealed no effect of high expression of EGFR on survival when compared to other biomarkers (p=0.367) or known prognosticators for recurrence (p=0.161).

### p53 expression

High p53 expression was evident in 38.4% of tumour specimens. There was no association between p53 expression and nodal disease at presentation (p=0.092) or development of distant metastatic disease (0.561). Kaplan-Meier analysis demonstrated significantly decreased RFS with high expression of p53 (p=0.005, Table 2, Figure 2). Cox regression analysis revealed a significantly decreased RFS when compared to other biomarkers (p=0.021, Table 3), but the relationship became non-significant when compared to known prognosticators for recurrence and p16 expression (p=0.129, Table 4).

### Bcl-XL expression

High expression of Bcl-xL was demonstrated in 47.3% of tumour specimens. High Bcl-xL expression was associated with positive nodal disease at presentation (p=0.039), but not with development of distant metastases (p=0.852). Combined biomarker analysis revealed a loose association of Bcl-xL expression with p16 expression (p=0.086). Kaplan-Meier analysis showed no effect on RFS. Cox regression analysis demonstrated no significant effect on RFS when compared to other biomarkers (p=0.231) or known prognosticators for recurrence (p=0.111).

## Discussion

There is significant variability and ambiguity in the literature surrounding the role of biomarkers in locoregional and distant metastasis. This is due, in part, to a non-standardized methodology in the immunohistochemical staining and quantification of biomarkers for each tumour specimen and from each institution. For example, immunohistochemical staining levels between slides within the same sample may have some variability in staining intensity, and pathologists may be limited to pseudo-quantitative methods in the interpretation of signal intensity. In addition, various antibodies have been used by different research groups for different biomarkers, with variable interpretations of signal positivity that does not take into account differences in nuclear versus cytoplasmic staining. Such biases may be overcome using TMAs with standardized, automated and quantitative immunohistochemical techniques, such as AQUAnalysis®. The objectivity of the AQUAnalysis® method allows for an unbiased assessment of biomarker staining. Bose et al. [10] demonstrated superior methodology in a study using AQUAnalysis® to correlate the expression of Bax, Bcl-2, and Bcl-xL in oral cavity squamous cell carcinoma specimens with disease-specific survival, and demonstrated reliable and reproducible estimates of expression for each biomarker.

In the present study, we have confirmed a previously demonstrated finding wherein p16 positivity can be strongly correlated with a higher incidence of nodal disease at presentation. Shonka Jr et al. [11] conducted a study in 69 OPSCC patients treated with primary radiotherapy, with or without chemotherapy, and post-treatment neck dissection. This study demonstrated that patients with p16-positive tumours presented with more advanced locoregional disease - a finding consistent with the results of our study. However, we have also demonstrated that nodal disease at presentation can be correlated with high Bcl-xL expression.

Bcl-xL is an anti-apoptotic protein in the Bcl-2 family that has recently stimulated interest in the head and neck cancer literature as a prognostic marker. However, its specific role in lymph node metastases has not been elucidated. Previous studies have demonstrated that high Bcl-xL expression can be associated with chemoradiation resistance [12,13]. A recent study demonstrated that high Bcl-xL expression in conjunction with high p53 expression can produce Cisplatin resistance in vitro [14]. Furthermore, a study of 50 patients by Kumar et al.^iii^ revealed that high Bcl-xL expression alone was not associated with treatment response or survival outcomes, but low expression levels in combination with low expression levels of p53 had higher overall and disease-specific survival than alternative combinations, potentially supporting the previous findings of chemoradiation resistance in specimens with high levels of both Bcl-xL and p53. No previous studies have examined the association of Bcl-xL with locoregional metastases. Our study demonstrates that high expression of Bcl-xL in OPSCC tumour specimens is associated with positive nodal disease at presentation. Although high expression of both p16 and Bcl-xL was associated with nodal disease at presentation, no correlation could be found between the biomarkers in a combined analysis, suggesting a dichotomous pathway for nodal spread wherein may Bcl-xL is associated with the carcinogenically-mediated, and p53-associated, OPSCC or an entirely different pathway of tumorigenesis as opposed to the virally-mediated pathway involving p16 expression. Combined biomarker analysis did not demonstrate a significant association between Bcl-xL and p53 expression in our study, suggesting that the latter explanation may be the most accurate.

Our study showed that p53 was not correlated with locoregional lymph node metastases or the development of distant metastases. However, the consistency of association with higher recurrence rates with high p53 expression is appropriate in the context of a dichotomized tumorigenesis pathway involving the distinction between carcinogenically-mediated OPSCC and virally-mediated OPSCC, wherein high expression of p53 is often detected in carcinogenically-mediated, p16-negative tumors. Our demonstration of an inverse relationship involving high p16 expression and low p53 expression in combined biomarker analysis supports this dichotomy. Increased incidence of p53 mutation in the setting of continued carcinogenic insult supports a finding of increased local recurrence as a result of adjacent and satellite chromosomal aberration and cellular dysplasia. Despite this, although studied extensively, p53 has not demonstrated utility in predicting lymph node metastases in previous studies [15-19]. High levels, however, have been shown to be associated with decreased overall survival, likely as a result of recurrence as previously described [20]. Again controversy exists regarding this claim, as a systematic review by Tandon et al. [21] failed to show any prognostic utility of p53 in predicting response to treatment.

Similarly, despite extensive study, ambiguity surrounds the role of EGFR in lymph node metastases. Due to the intricate and extensive role of EGFR in signal transduction, aberrant EGFR function has potential to affect cell cycle progression, apoptosis, and angiogenesis [22], and high expression has been shown to be associated with poor prognosis [23-25], yet this has been contended with several opposing studies [26,27]. Despite high overall expression levels of EGFR positivity in our cohort, EGFR was not shown to be predictive of nodal disease at presentation or of development of distant metastatic disease. Although high expression of EGFR is anticipated in HNSCC, lack of literature consistency regarding its effect on metastasis and survival may be due to diverse staining and isolation techniques, different quantitative biomarker thresholds, or a lack of objective or automated biomarker quantification. In addition, as multiple EGFR mutations exist, and multiple aberrations of the downstream intracellular pathways exist, further study is required to assess the optimal form, and dysfunction, to measure regarding anomalies in the EGFR pathway.

Ki67 is a relatively novel biomarker of nuclear proliferation recently examined in the head and neck literature. In a previous study by Boonkitticharoen et al. [28], manual scoring technique was used to assess the degree of positivity of oral and oropharyngeal SCC in association with low expression of vascular endothelial growth factor (VEGF). This demonstrated that low VEGF and high Ki67 levels were associated with lymph node metastases. However, our study found no significant association with nodal disease at presentation, the development of distant metastases, or recurrence. Explanations for the discrepancy in findings likely relate to diverse methods of immunostaining and immunohistochemical isolation (particularly of concern given its ubiquitous existence in the cell), differences in biomarker cutpoints, variation in the density of Ki67 expression depending on the cellular or tumor region examined (eg. nucleus or cytoplasm), or a difference in expression related to anatomic site. For example, Klimowicz et al. [29] used AQUAnalysis® to show that high basal Ki67 expression is associated with improved prognosis in oral cavity SCC patients receiving postoperative radiation therapy, whereas Nichols et al. [30] demonstrated a higher rate of recurrence of early glottic cancer with high Ki67 expression when treated with radiotherapy.

There were several limitations in our study. This was a retrospective study of a prospectively-collected database of OPSCC patients from a single institution. Despite sound quantitative analysis of biomarkers, a number of samples were excluded due to an inadequate amount of tumour tissue. This could be reduced with prospective tumour banking and continued quantitative biomarker analysis using AQUAnalysis.

The process of metastasis is complex. Factors that promote metastasis of cancer cells involve alterations in genes and gene products at multiple stages in the cell cycle and signal transduction pathways. Thus, in order to determine utility in predicting locoregional or distant metastases, multiple biomarkers must be evaluated, and potential independent and combined function and dysfunction deciphered. Further study regarding the optimal form of each biomarker to be measured, and the optimal method of measurement of each biomarker should be undertaken. Additionally, standardized and automated quantitative immunohistochemistry should be utilized in order to adequately compare findings between institutions and ultimately, allow for individualization and optimization of treatment by reducing toxicity.

## Conclusions

This study supports previous findings that high expression of p16 is associated with a higher incidence of nodal disease at presentation, but a higher recurrence-free survival. However, this study has also demonstrated that high Bcl-XL expression is associated with nodal disease at presentation, but that none of the biomarkers can be used as a reliable prognostic marker for distant metastases. Lastly we have demonstrated that an inverse relationship exists between p16 and p53 expression, that high p53 expression may be useful as a prognostic factor for recurrence in head and neck cancer, but that further studies are necessary to assess the prognostic value of biomarker profiling in OPSCC.

## Abbreviations

RFS: Recurrence-free survival; EGFR: Epidermal growth factor receptor; Bcl-xL: B cell lymphoma extra large; HPV: Human papillomavirus; OPSCC: Oropharyngeal squamous cell carcinoma; HNSCC: Head and neck squamous cell carcinoma; PCK: Pan cytokeratin; TMA: Tissue microarray.

## Competing interests

The authors declare that they have no competing interests.

## Authors’ contributions

Dr. BB contributed with data collection, statistical analysis, and manuscript preparation. Dr. VB contributed with manuscript preparation and revision, as well as study design. Dr. LP provided insight regarding biomarker roles, and assisted with manuscript revision. Dr. AK prepared and analyzed all TMAs, and assisted with data collection. Dr. DC assisted with study design and manuscript revision. Dr. HS assisted with study design, manuscript preparation, and manuscript revision. All authors read and approved the final manuscript.
